# Characters matter: How narratives shape affective responses to risk communication

**DOI:** 10.1371/journal.pone.0225968

**Published:** 2019-12-09

**Authors:** Elizabeth A. Shanahan, Ann Marie Reinhold, Eric D. Raile, Geoffrey C. Poole, Richard C. Ready, Clemente Izurieta, Jamie McEvoy, Nicolas T. Bergmann, Henry King

**Affiliations:** 1 Department of Political Science, College of Letters & Science, Montana State University, Bozeman, Montana, United States of America; 2 Montana Institute on Ecosystems, Montana State University, Bozeman, Montana, United States of America; 3 Department of Land Resources & Environmental Sciences, College of Agriculture, Montana State University, Bozeman, Montana, United States of America; 4 Department of Agricultural Economics & Economics, College of Agriculture, Montana State University, Bozeman, Montana, United States of America; 5 Department of Computer Science, Gianforte School of Computing, Montana State University, Bozeman, Montana, United States of America; 6 Department of Earth Sciences, College of Letters & Science, Montana State University, Bozeman, Montana, United States of America; Indiana University Bloomington College of Arts and Sciences, UNITED STATES

## Abstract

**Introduction:**

Whereas scientists depend on the language of probability to relay information about hazards, risk communication may be more effective when embedding scientific information in narratives. The persuasive power of narratives is theorized to reside, in part, in narrative transportation.

**Purpose:**

This study seeks to advance the science of stories in risk communication by measuring real-time affective responses as a proxy indicator for narrative transportation during science messages that present scientific information in the context of narrative.

**Methods:**

This study employed a within-subjects design in which participants (n = 90) were exposed to eight science messages regarding flood risk. Conventional science messages using probability and certainty language represented two conditions. The remaining six conditions were narrative science messages that embedded the two conventional science messages within three story forms that manipulated the narrative mechanism of character selection. Informed by the Narrative Policy Framework, the characters portrayed in the narrative science messages were hero, victim, and victim-to-hero. Natural language processing techniques were applied to identify and rank hero and victim vocabularies from 45 resident interviews conducted in the study area; the resulting classified vocabulary was used to build each of the three story types. Affective response data were collected over 12 group sessions across three flood-prone communities in Montana. Dial response technology was used to capture continuous, second-by-second recording of participants’ affective responses while listening to each of the eight science messages. Message order was randomized across sessions. ANOVA and three linear mixed-effects models were estimated to test our predictions.

**Results:**

First, both probabilistic and certainty science language evoked negative affective responses with no statistical differences between them. Second, narrative science messages were associated with greater variance in affective responses than conventional science messages. Third, when characters are in action, variation in the narrative mechanism of character selection leads to significantly different affective responses. Hero and victim-to-hero characters elicit positive affective responses, while victim characters produce a slightly negative response.

**Conclusions:**

In risk communication, characters matter in audience experience of narrative transportation as measured by affective responses.

## Introduction

Humans are innate storytellers or *homo narrans* [1]. What water is to fish, stories are to humans. Stories or narratives surround us in ways that are both vital to our existence and yet often go unnoticed. Humanist scholars conceptualize narratives as fundamental expressions of culture, identity, and values [2]. More recently in the social sciences, scholars are investigating the power of narratives to influence perceptions and behaviors [3, 4]. However, as central as stories are to the human condition, scientific writing and communication have been intentionally developed as non-narrative, parsimonious, and objective [5]. In the realm of hazards such as floods, earthquakes, and wild fires, conventional risk communication efforts are often ineffective at inducing people’s preparedness [6, 7]. One possible explanation is that scientists and the public do not share a common language to describe risk. Scientists tend to rely on the language of probability, uncertainty, frequency, and magnitude, whereas most people communicate their realities through stories, replete with characters, plotlines, and settings [8]. Not surprisingly, risk communication studies generally find narrative language more effective than science-only language at influencing factors such as risk perception and behavior [9, 10], yet the role and efficacy of various narrative mechanisms remains largely unstudied. An improved understanding of how specific narrative mechanisms function to influence and persuade the audience may improve risk communication efforts.

## Narrative mechanisms, risk communication, and affect

### Narrative mechanisms

Scientists model social, natural, and physical phenomena by identifying how specific mechanisms function to produce these phenomena [11]. If persuasion in narrative risk communication is the phenomenon, the mechanisms conceptualized to induce persuasion are found in narrative. The Narrative Policy Framework (NPF) [12] provides a theoretical foundation for identifying and defining narrative mechanisms. Informed by narratology [13], the NPF conceptualizes narrative as having structure that includes narrative elements such as characters, plot, and setting. For example, many stories take place in a setting where a hero’s actions follow some plot to save a victim from a villain. According to the NPF, narrative structure in storytelling is universal and results in the ability to analyze narratives across time and space for different policy issues. As such, identifying narrative mechanisms to test narrative effects in risk communication can be anchored in the NPF’s narrative elements.

The NPF describes characters as human and non-human entities engaging in the action of the story [14] and assumes that at least one character must be present in a narrative [15]. Therefore, characters are a logical place to focus a study of the effects of narrative mechanisms. While there is no universal character typology, the most common characters are heroes, victims, and villains [12]. The NPF defines heroes as the fixers of the problem, victims as those who suffer or fear harm from a problem, and villains as those who cause the problem [12, 16]. NPF researchers have consistently found that narratives featuring a hero are effective in shaping individual opinion [17], but the full causal pathway from hero to opinion change is understudied. Additionally, much of the content of extant narrative risk communication is based on presentation of harm, intended to activate fear to motivate people into action [18]; unlike studies centered on the hero, the NPF has not deeply examined the power of victim characters or the idea of victim turns hero. In sum, selection of character types in the action of a story is an important narrative mechanism.

### Narrative transportation

Social psychologists link the persuasive power of narratives to narrative transportation [19, 20], a process that happens when the audience is absorbed into a story such that they are lifted out of the realities of their lives and actually feel the story experience. Thus, by definition, narrative transportation is partly an affective experience of the narrative as it unfolds. Using an exploratory factor analysis, Green and Brock identify three dimensions of narrative transportation from a 15-item questionnaire: cognitive, emotional-affective, and visual imagery [19, 21]. Cognitive transportation is the extent to which the story holds one’s attention; affective response is one’s positive or negative response to the story; visual imagery is how vividly one pictures particular characters in the story [19–22]. Understanding the extent to which a narrative mechanism—such as the selection of a hero or victim character—stimulates affective responses, and thus narrative transportation, can ultimately lead to explanatory hypotheses about the roles and power of narrative mechanisms in narrative persuasion.

To quantify narrative transportation, scholars consistently employ a *post hoc* questionnaire after exposure to some narrative text [19]. As with any post-treatment subjective evaluation, the responses reflect one point in time and require retrospective evaluation. While this measure has offered insights into narrative transportation, the experience of a narrative also occurs over the time of narrative exposure, not just after the story. Thus, measuring the audience’s experiences in real time will offer a more precise understanding of how narrative mechanisms function.

### Risk communication and affect

The goal of hazard communication is to reduce vulnerability by closing the gap between scientific prediction of risk and the public’s perception of the same risk [23, 24]. Yet conventional risk communication is predominantly comprised of the latest scientific information that, in isolation, is often ineffectively assimilated into people’s hazard preparedness [6, 7]. Consequently, a growing number of risk scholars have examined the inadequacies of conventional risk communication. Some scholars test the effects of scientific data displays such as maps [23, 25]. Others explore social processes involving risk information exchange, communication venues, and trust of science [26, 27]. Still others examine individual cognitive processes that shape risk perception [28], including cultural theory [29], cognitive biases [30], and economic incentivizing [31]. Notably, Slovic’s and Loewenstein’s voluminous work [32–40] examining the role of affect or emotional responses in risk assessment is particularly influential, suggesting that affective responses can change how audiences perceive risk communication and will thus result in changes to risk perceptions and decisions.

Slovic and Loewenstein posit that a stimulus (e.g., receiving a form of risk communication) that evokes an affective response (i.e., positive or negative feelings) is more likely to influence individual assessments of risk and ensuing behavior than are objective facts [32–34, 36, 38–42]. In other words, feelings associated with risk influence an individual’s cognitive judgments about the likelihood and severity of a hazard. These findings have led to greater attention being paid to characteristics of risk communication stimuli or messages themselves [42], such as use of frames [43], advocacy for compelling visualizations [44], and documentary films [45]. All of these studies share a notion of the importance of some kind of rhetorical communication device or imagery or story to evoke an affective response.

Whereas narratives are largely understood as powerful in their ability to persuade people [46], the study of narratives in risk communication is nascent. In the health domain, scholars [9, 47] resoundingly find stronger changes in risk perception and behavior when using “personal accounts” as opposed to science messages. Narrative risk communication scholarship [24] tends to examine the relative impacts of technical versus narratively presented information. However, the narrative constructions in such studies are problematic because they are *ad hoc* constructions of stories made up by scientists or researchers. In other words, these studies find that narratives influence risk perceptions and reported decisions, but the mechanisms involved in narrative persuasion are neither clearly identified nor understood. Greater precision in examining narrative mechanisms (e.g., selection of types of characters) is necessary if we wish to more accurately understand the narrative effects of communicating scientific information. Further, linking affective responses to narrative mechanisms is needed to understand more precisely how these mechanisms function.

## Current study

This study proposes to advance the science of stories in risk communication by measuring real-time affective responses to conventional science messages and narrative science messages. Similar to other studies measuring the valence of participant affect [48–50], we capture affective experience with dial response technology that records participants’ second-by-second positive and negative responses as they hear the conventional and narrative science messages. Affect is a distinct dimension of narrative transportation [19–22] that has not been measured “in transport” or over time while experiencing risk communication messages. When exposed to the narrative mechanism of character selection, we consider the magnitude of affective response—regardless of direction (positive or negative)—to indicate the magnitude of narrative transportation.

We also seek to advance the understanding of affective responses to differently framed conventional science messages. Used as control conditions, these messages are comprised of a definition of the hazard and a description of the risk using the scientific language of either probability or certainty. Science information is typically communicated using the language of probability (e.g., odds of something happening) and remains largely unclear to the general public [51]. However, an emerging practice in risk communication uses the language of certainty to discuss hazards (e.g., a hazard event will occur at some point) to nudge people towards better hazard preparation decisions [52, 53]. In this study, we test affective responses to conventional science messages that include a definition of flooding and that communicate science information in the language of probability (i.e., the chance of a large flood occurring) or the language of certainty (i.e., a large flood will occur at some point in the future). Since probability and certainty statements are non-narrative stimuli to which the target audience may have affective responses, we employ *transportation* to describe responses to statements that include non-narrative language; we use *narrative transportation* to describe responses to statements that include only narrative language.

Finally, we bring precision to the study of narratives as a treatment in risk communication studies by: 1) maintaining science language in the narrative text so as to isolate narrative effects; and 2) developing the narrative text to test only the selection of character type using “hero” and “villain” vocabulary employed in the communities where test subjects reside.

## Research questions and predictions

Research question 1. Do affective responses to probabilistic language differ from affective responses to certainty language?

Earthquake hazard communication is often couched in certainty language [52, 53] (i.e. an earthquake will happen at some point), yet flood hazard information is still typically presented in terms of probabilities [54] (e.g., a flood of a certain magnitude will occur, on average, once every 100 years). We explore whether presenting scientific flood hazard information in certainty vs. probability language results in differing affective responses.

Prediction 1: Certainty language will produce larger affective responses than will probability language within science messages.

Research question 2. Does narrative language influence affective responses to hazard preparedness messages?

Risk communication studies find that personal narratives are more effective at persuasion than conventional science messages [9], and the narrative transportation literature suggests that persuasive narrative messages emotionally engage respondents [19, 20]. Integrating these two bodies of literature, we explore whether transportation as measured through affective responses differs between conventional science messages and narrative science messages.

Prediction 2a: Affective responses will vary more over the course of narrative science messages than over the course of conventional science messages.

More precisely, we explore the affective responses to the narrative mechanism of character selection. We test whether the type of character presented in the narrative evokes varying levels of positive and negative affect. Since NPF studies suggest that people respond differently to narrative messages depending on which characters appear in the message [55], we examine the narrative mechanism of characters deployed in the story.

Prediction 2b: The direction and magnitude of affective responses will depend on the narrative mechanism of character type selection (i.e., hero, victim, and victim-to-hero).

## Flood risk on the Yellowstone River

**Fig 1:**
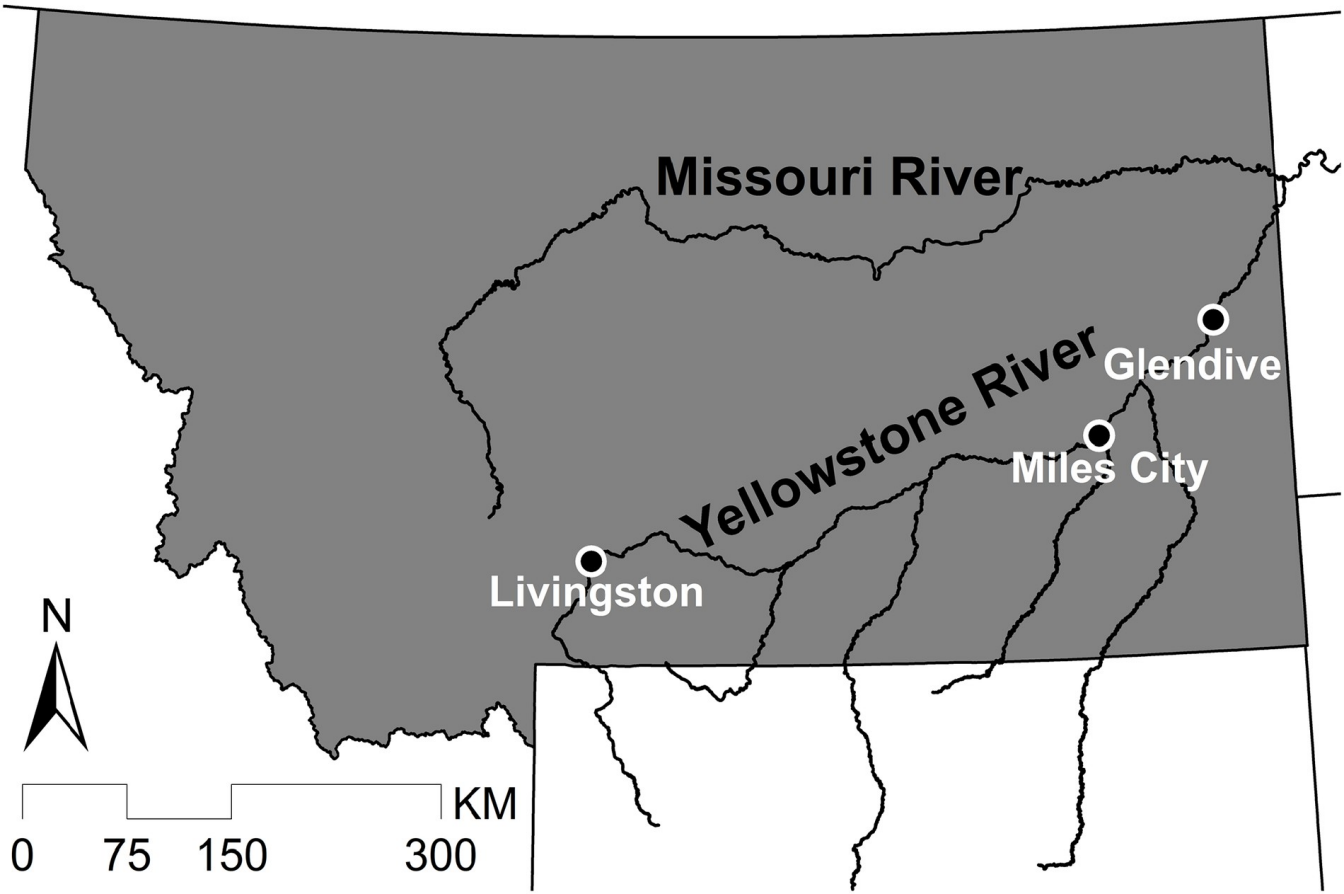
Map of Montana, USA, with Yellowstone River and three study communities.

To test the extent of transportation for conventional science messages and narrative science messages, we focus on flood risk along the Yellowstone River in Montana. The Yellowstone River flows ~1100 km from its source in northwestern Wyoming to its confluence with the Missouri River in western North Dakota (Fig 1). The Yellowstone River poses flood threats to communities along the river [56, 57]. The present study involves discussion of flooding in three of these Montana communities–Livingston, Miles City, and Glendive. Livingston, known for its proximity to Yellowstone National Park and its world-class trout fishing, experiences spring flooding that threatens commercial and residential buildings as well as its recreation economy. Miles City, located at the confluence of the Tongue River and Yellowstone River, responded to historic floods in the 1920s and 1940s by installing a dike or levee system. However, that system is not currently certified by the Federal Emergency Management Agency (FEMA), resulting in many residents living in the designated 100-year floodplain [58]. Glendive, located just 90 miles upstream from the Yellowstone’s confluence with the Missouri River, also has a levy built in the 1950s; like Miles City, this levy is not certified, resulting in difficulties with business development in an area designated as 100-year floodplain [59].

## Methods

This study employs a within-subjects design in which participants were exposed to eight risk message conditions: two conventional science messages and six narrative science messages. We collected the data over 12 group sessions across the 3 study communities. Randomizing the order of the science messages across each session mitigated order effects.

This human subjects research was approved by Montana State University IRB # ES021616-EX.

### Construction of science messages

#### Conventional science messages

Conventional science messages are comprised of two parts: (1) *Flood definition* and (2) *Science information*. The *Flood definition* is anchored in material from FEMA [60] as well as from the hydrology experts on the research team; the same *Flood definition* is replicated in all science messages. *Science information* uses one of two approaches to talk about flood risk, either *Probability* or *Certainty* language.

*Probability* language is based on terms used to describe flood frequency analysis (FFA) of historic river flow data, the most common method in the United States to assess flood risk. In the parlance of FFA, a “100-year flood” is associated with a river discharge rate that has been exceeded, on average, once in a 100-year period. Yet, using such a term can result in a subtle understatement of flood risk because the terms ignore the fact that probability of a flood increases with the time period considered. For instance, although river discharge associated with a 100-year flood has only a 1% chance of occurring in any given year, there is a 22.2% chance of such an event occurring each generation (25 years) and a 54% chance of such an event occurring in the average lifetime of a baby born in the U.S. (78 years). The *Probability* language in the science messages is designed to highlight this aspect of flood frequencies, using a 30-year period—the typical length of a home mortgage.

*Certainty* language is designed to mimic the way some scientists communicate earthquake risk [53]. The language states that large floods will happen in the future, even if we do not know exactly when. The *Certainty* language used in this study was based on the idea that Holocene river sediment deposits indicate where floods have happened over the last 10,000 years [61] and are, therefore, an indication of areas where floods will happen again.

#### Narrative science messages

To develop valid narrative science messages, we began by discovering who the relevant characters are in the lives of the target population. We interviewed 45 individuals across the 3 study communities between February and June 2017 (S1 Protocol. Interview protocol), with interview length ranging from 25 minutes to over 2 hours. Using NVivo11 software [62], researchers independently human-coded the interviews for narrative characters, using an abbreviated version of an NPF codebook that provided definitions for character nodes and coding instructions (S1 Codebook. Narrative Policy Framework codebook) [15]. The specific identities of these characters (i.e., the subnodes within the hero and victim character parent nodes) emerged inductively from the data (e.g., hero_government floodplain administrator or hero_levee). Twenty percent of each interview was randomly selected to check for intercoder reliability. Averaging across all interviews, we find strong intercoder reliability [63] with a Cohen’s kappa for “hero” coding equal to .8829, and for “victim” coding equal to .8796.

Since we coded at the sentence level, the amount of character-related text was substantial (e.g., the hero corpus contained ~35,000 words; the victim corpus contained ~58,000 words). We then employed natural language processing (NLP) to rank the frequency of specific words used within the context of “hero” and “victim” coded language. Given our population of interviews (*I*), we divided each interview (*i*) into two documents; the first document contained only hero-coded language from interview *i* and the second contained only victim-coded language from interview *i*. We converted the text of each document to lowercase, and removed all numbers, punctuation, and stop words (i.e., commonly used words like “and” and “the”) from the documents. Remaining text from all hero documents was aggregated into the “hero corpus,” and remaining text in the victim documents formed the “victim corpus.” We calculated the frequency (*n*) for of each word (*w*) in each document (*n*_*w*,*i*,*c*_, where *c* represents a corpus). From the *n*_*w*,*i*,*c*_ values, we calculated a frequency rank (a transformed relative frequency, or *TRF*) for each word in each corpus (*TRF*_*w*,*c*_). *TRF*_*w*,*c*_ was the square root of *n*_*w*,*i*,*c*_ value for each word, summed across interviews for each corpus, divided by the total number of words in the corpus (*n*_*c*_), and rescaled by a factor of 10,000:

$${TRF}_{w,c}=10,000\frac{\sum_{i=1}^{I}\sqrt{n_{w,i,c}}}{n_{c}}$$ (1)

A square root transformation is used in the numerator of Eq 1 to reduce the effect of individual interviewees sometimes repeating a word many times. The scaling factor of 10,000 simply converted values on the order of 0.00001 to 0.01 to more intuitive values on the order of 0.1 to 10. Given that some words appeared in both the hero corpus and the victim corpus, we subtracted *TRF*_*w*,*victim*_ values from *TRF*_*w*,*hero*_ values to obtain the final *TRF* value for each word (*TRF*_*w*_) occurring in either corpus:

$${TRF}_{w}={TRF}_{w,hero}-{TRF}_{w,victim}$$ (2)

In this way, a word with identical *TRF* values in both the hero and victim corpora would have a final *TRF*_*w*_ value of zero. Positive *TRF*_*w*_ values represent words appearing only in the hero corpus *or* words that ranked higher in the hero corpus than in the victim corpus. Negative values represent the opposite. We ranked all words by their *TRF*_*w*_ values and took the top 4% of words as the “hero vocabulary” and the bottom 4% of words as the “victim vocabulary.”

Drawing from these vocabularies, we constructed three narrative texts for the narrative mechanism of character selection: *Hero narrative*, *Victim narrative*, and *Victim-to-hero narrative*. The *Hero narrative* emphasizes that audience members and their communities are capable of preparing for flood-related problems, while using words from the hero vocabulary and avoiding words in the victim vocabulary. The *Victim narrative* emphasizes negative outcomes for the audience members and their communities, while using words from the victim vocabulary and avoiding words in the hero vocabulary. The *Victim-to-hero narrative* creates an arc in which the negative outcomes can be reversed by the audience members and their communities, preferring words from the victim vocabulary in the beginning and words from the hero vocabulary toward the end.

To construct the narrative science messages, we embedded the two conventional science messages (*Flood definition* and either *Probability* or *Certainty* language for *Science information*) within the three narratives featuring different character types (S1 Text. Science messages with segments identified). The result is that the narrative science messages are comprised of four *Segments*: *Flood definition*; *Problem framing (Hero*, *Victim*, or *Victim-to-hero)*; *Science information (Probability* or *Certainty* language); and *Characters in action (Hero*, *Victim*, or *Victim-to-hero)*. In contrast, the conventional science messages contain only two *Segments*: *Flood definition* and *Science information* (Table 1).

**Table 1:** Message construction.

Scientific language	Science message type (duration in seconds)	Segment			
		Flood definition^a^	Problem framing^b^	Science information^c^	Characters in action^d^
Probability	Conventional (46)	FD		SI_P_	
	Hero narrative (91)	FD	PF_H_	SI_P_	CiA_H_
	Victim narrative (90)	FD	PF_V_	SI_P_	CiA_V_
	Victim-to-hero narrative (94)	FD	PF_VtoH_	SI_P_	CiA_VtoH_
Certainty	Conventional (41)	FD		SI_C_	
	Hero narrative (93)	FD	PF_H_	SI_C_	CiA_H_
	Victim narrative (95)	FD	PF_V_	SI_C_	CiA_V_
	Victim-to-hero narrative (99)	FD	PF_VtoH_	SI_C_	CiA_VtoH_

^a^Flood definition is the first segment of the science messages; FD denotes the language used to define a flood hazard.

^b^Problem framing is the second segment of the science messages, with PF_H_ = the language of problem framing in the hero narrative; PF_V_ = the language of problem framing in the victim language; PF_VtoH_ = the language of problem framing in the victim-to-hero narrative.

^c^Science information is the third segment of the science messages, with SI_P_ = the science information of flood risk presented in probability language; SI_C_ = the science information of flood risk presented in certainty language.

^d^Characters in action is the fourth segment of the science messages, CiA for _H_, _V_, and _VtoH_ denoting the use of hero, victim, and victim-to-hero as the characters.

The *Problem framing* segment introduces the characters and identifies the problem of extreme flooding. Locally derived language was used to develop the text for the problem frame, which indicated that under extreme flood conditions the river could “over-top” the community’s levee or bank.

The *Characters in action* segment is the part of the narrative text where the characters provide the action of the story. For any risk communication, the desired action is for people to prepare for an impending disaster. In this study, we were intentionally vague about the hazard preparation behavior itself as we sought to avoid evoking affect associated with a particular decision. For example, the *Hero* in action language refers broadly to strategies:

“Working with your local emergency responders, you can think about and begin to implement individual and community strategies before a disaster occurs. By trying to protect from damages of extreme flooding, you will have really helped in a big way.”

The *Victim* is confronted with the consequences of no preparation:

“You, your friends, and your neighbor could be harmed or wiped out by high post-flood premiums and loss of valuable assets such as cattle and houses. Without preparation, your town could be lost as it faces difficult and sad times.”

The *Victim-to-hero* language starts with the victim not preparing and turning into the hero by implementing preparation strategies.

“Without preparation, your town could be lost as it faces difficult and sad times. Working with your local emergency responders, you can think about and begin to implement individual and community strategies before a disaster occurs.”

In all, the study contains two conventional science messages (representing *Probability* vs. *Certainty* language in the *Science information* segment) and six narrative science messages (three character types crossed with *Probability* vs. *Certainty* language) (Table 1).

### Measuring affective responses

**Fig 2:**
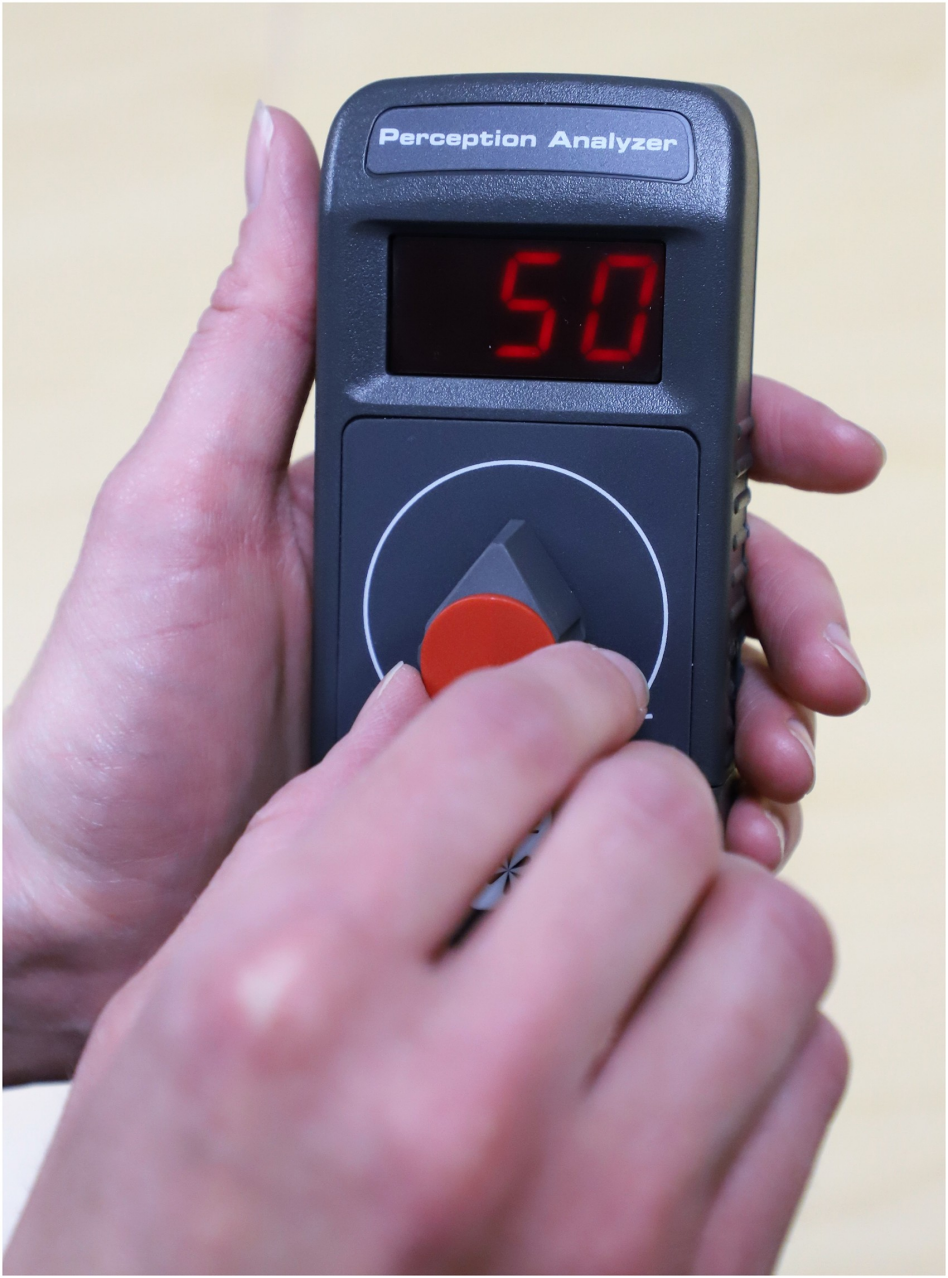
Dial device used to collect second-by-second affective responses.

To quantify transportation, we used the Perception Analyzer^®^ from Dialsmith [64] to instantaneously record (once per second) participant affective responses to the eight science messages. Participants held dials with a data range from 0 to 100, with the vertical position of the dial at 50 (Fig 2). The facilitator trained the participants to turn the dial toward 100 when they felt positive about what they were hearing and to turn the dial toward 0 when they felt negative about what they were hearing. These dial readings (as seen at ‘50’ in Fig 2) were visible to each participant as they turned their dial. The verbatim instructions stated: “We ask that you start with your dial in the straight-up position, which is neutral. As we go through each message or story, you adjust the dial upward or to the right when you like what you are hearing. Similarly, you adjust the dial downward or to the left if you do not like what you are hearing. You can adjust the dial continuously throughout the entire message.” Therefore, a dial reading of 50 does not indicate neutral affect but a point at which participants have equal opportunity to turn the dial up or down (baseline); in fact, one study found that the valence of affective response across time via dial movement was unaffected by starting point [65]. Therefore, based on our instructions to participants, we interpret positive or negative dial movement as an associated change in affective response. For consistency, the facilitator asked participants to reposition the dial to the start position of 50 at the onset of each science message.

To ensure that the delivery of each science message was the same for all participants, we put the eight science messages into an auto-play format in Microsoft PowerPoint with audio overlay. Each slide contained a single sentence from the message. The order of messages was randomized for each group of participants.

### Participants

We obtained a non-probability sample through multiple methods: mailing a postcard invitation to a random sample of 500 addresses for each of the 3 study communities, advertising in local newspapers and social media linked to local governments, and using a snowball technique from those recruited via postcards or advertisements. A $50 incentive was offered to participants. The research sessions took place over a 3-week period late in 2017 in the 3 study communities where the initial interviews were conducted. The research team held 4 group sessions in each community, with 4 to 11 participants in each session. The final sample included 90 research participants: 36 from Livingston, 22 from Miles City, and 32 from Glendive. Of the 90 participants, 4 were excluded from all analyses because the dial data indicated they did not follow instructions; the average standard deviation of their dial reading was less than 1, meaning they did not turn their dials in response to the messages. A short questionnaire collected demographic information after the presentation of all science messages. The sample was nearly evenly split between women and men but did skew somewhat older (Table 2).

**Table 2:** Demographics.

	Participant Sex			Participant Age (Years)		
	Female	Male	No Reply	Mean	Range	Std. Dev.
Glendive (n = 31)	39%	61%	0%	58	22–85	15.8
Livingston (n = 35)	60%	40%	0%	57	27–83	15.4
Miles City (n = 20)	50%	45%	5%	60	25–76	13.2
Overall (n = 86)	50%	49%	1%	58	22–85	14.9

### Data analyses

All data management, plots, and statistical analyses were conducted in the R statistical computing environment [66].

#### Data visualization

The data for our analyses come from each participant’s second-by-second dial readings for each of the eight science messages. We plotted averaged dial readings across all participants at each point in time within each of the four *Segments* (*Flood definition*, *Problem framing*, *Science information*, and *Characters in action*) to visualize the transportation arc induced by each science message. Because no meaningful differences exist between measures of transportation between the *Scientific language* of *Probability* and *Certainty* (see Results: Prediction 1), we averaged second-by-second dial readings within each *Segment* for each *Science message type*. Primarily due to small differences in the length of *Probability* vs. *Certainty* language, the duration of *Segments* varied across messages by 1 to 8 seconds. Therefore, we plotted averaged dial readings against the elapsed percentage of each *Segment’s* duration (0 to 100%) to visually compare changes in dial readings across messages. In resulting plots, transportation is depicted by the upward (positive) and downward (negative) trails of mean dial readings across participants.

#### Data and measures of transportation

We measure transportation by the extent to which participants turn their respective dials either up or down, indicating positive and negative affective responses. We constructed three measures of transportation for each participant from the second-by-second dial readings for each science message. Our first indicator of transportation was variation in affective responses across conventional and narrative science messages, analyzed using the standard deviation (*S*.*D*.) of the dial readings for the entire message. *S*.*D*. is correlated with other measures of affective response such as range (*r* = .907; *p* < 2.2e-16; *t* = 56.432, *df* = 686) and total dial movement (*r* = .595; *p* < 2.2e-16; *t* = 17.921, *df* = 686). We chose *S*.*D*. because it prioritizes range of movement (S1 Fig Correlation plots). This prioritization is important given our assumption that larger swings in dial readings are indicative of greater absorption into the story and feeling of the story experience (i.e., transportation). Furthermore, unlike range and frequency, *S*.*D*. is standardized across message length thus allowing for comparisons between messages.

**Fig 3:**
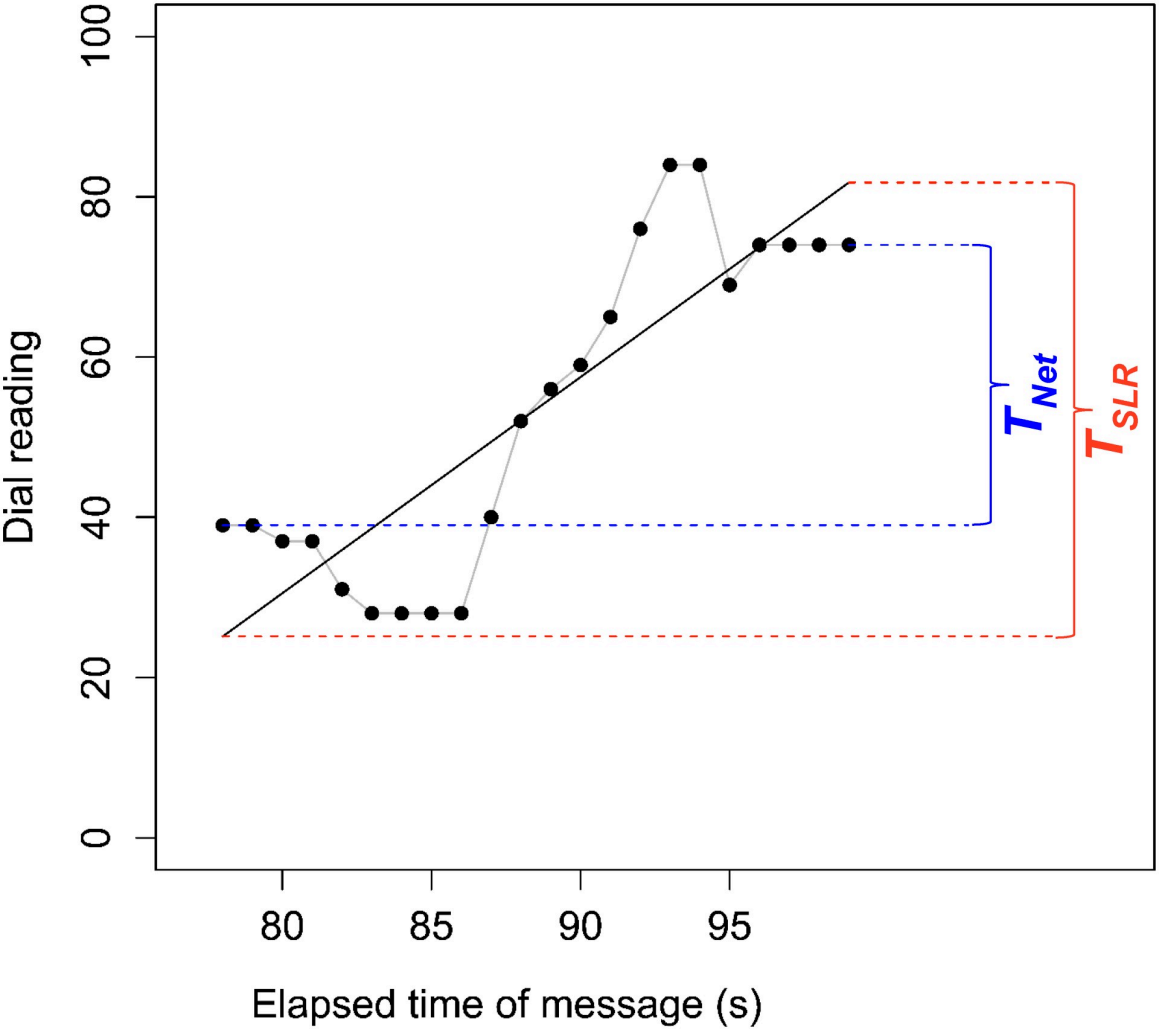
Illustration of how *T*_*Net*_ and *T*_*SLR*_ were calculated. Points represent the dial reading at each second for a participant during the *Characters in action* segment of the *Victim—to—hero* message with *Certainty* language. Gray line traces points. Solid black line denotes simple linear regression of dial reading against elapsed time of message. *T*_*Net*_ was calculated by subtracting the participant’s initial dial reading from their final dial reading within a segment. *T*_*SLR*_ was calculated by multiplying the estimated change in dial reading per second (regression slope) by the duration of the segment in seconds.

Our second and third indicators provide information about transportation (*T* [Δ dial reading]) during each of the four *Segments* for each message for each participant (Fig 3). Our first indicator of *T* is *T*_*Net*_, which was calculated by subtracting a participant’s dial reading at the start of each segment from the participant’s dial reading at the end of the same segment. Our second indicator of *T* is *T*_*SLR*_, which was determined from the slope of a simple linear regression of dial reading against elapsed message time. *T*_*SLR*_ was the product of the regression slope (Δ dial reading/second) and segment duration (seconds). *T*_*Net*_ and *T*_*SLR*_ both measure transportation over a message segment. Importantly, transportation for each segment is understood to be the change in dial readings relative to the starting point in each segment. *T*_*Net*_ is a direct measure but could be sensitive to brief, anomalous dial readings at the beginning or end of a segment. *T*_*SLR*_ measures trend over the entire segment and is less sensitive to brief anomalies in the dial readings. The two indicators are highly correlated (*r* = 0.876; *p* < 2.2e-16; *t* = 89.036, *df* = 2406). Both indicators of transportation are reported.

#### Statistical tests

To test our predictions, we estimated three linear mixed-effects models, with the dependent variables *S*.*D*., *T*_*Net*_, and *T*_*SLR*_. We deemed linear models without censoring to be appropriate because the data showed little evidence of ceiling or floor effects. Few of the dial readings were above 95 (6.7% of all readings) or below 5 (0.8%) on the 0–100 measurement scale. The model with *S*.*D*. as the dependent variable had fixed effects for *Science message type* (*Conventional*, *Hero*, *Victim*, and *Victim-to-hero*), *Science language* (*Probability*, *Certainty*), and *Town* (*Livingston*, *Glendive*, *Miles City*). The models with *T*_*Net*_ and *T*_*SLR*_ as dependent variables had fixed effects for *Science message type*, *Science language*, *Town*, *Segment* (*Flood definition*, *Problem framing*, *Science information*, and *Characters in action*), and the interaction of *Segment* and *Science message type*. In all models, a random intercept was fitted for each respondent because each respondent provided multiple responses.

Prediction 1. An analysis of variance was used to test whether affective responses differed according to the *Scientific language* (*Certainty* vs. *Probability*) embedded in each *Science message type* for the *S*.*D*., *T*_*Net*_, and *T*_*SLR*_ models.
Prediction 2a. The linear mixed-effects model with *S*.*D*. as the dependent variable was used to test whether narrative science messages induced more affective response than conventional science messages. Two tests were conducted. First, we used an analysis of variance to test whether *S*.*D*. of message scores differed among *Science message types*. Second, pairwise comparisons of the estimated marginal means for each *Science message type* were calculated to determine differences in levels of variation in affective response scores.
Prediction 2b. The linear mixed-effects models with *T*_*Net*_ and *T*_*SLR*_ as dependent variables were used to test whether transportation within a *Segment* varied depending on the *Science message type*. Two tests were conducted for each model. First, we used an analysis of variance to test whether transportation within each *Segment* differed among *Science message types*. Second, pairwise comparisons of the estimated marginal means for each *Science message type* were calculated for each of the four *Segments* to determine which *Science message types* induced more positive or more negative affective responses within each message *Segment*.

Each model was fit using the *lmer* function from the lme4 R package version 1.1.21 [67]. Tests for the significance of fixed effects were conducted using the *anova* function from the lmerTest R package version 3.1.0 [68] using Kenward-Roger approximations for degrees of freedom per the findings of Luke [69] and α = 0.05. Using the emmeans R package version 1.3.3 [70], point estimates of estimated marginal means with associated 95% confidence intervals were calculated and pairwise comparisons within factor levels were assessed with α = 0.05; all point estimates and pairwise-differences were calculated averaging across all other model terms.

## Results

### Prediction 1

In contrast to our prediction, we find no differences in measures of affective response between *Probability* and *Certainty* language. *Certainty* language does not induce larger variation in affective responses (see *Scientific language* in Table 3) or larger transportation in affective responses over a *Segment* than *Probability* language (see *Scientific language* in Table 4). The hypothesis that use of certainty language improves risk communication by influencing affective responses is not supported. Our results leave open the possibility that certainty language improves risk communication via other mechanisms.

**Table 3:** Analysis of variance results for standard deviations in affective response over the course of science messages.

	Sums of squares	Mean squares	Numerator degrees of freedom	Denominator degrees of freedom	*F*-value	*p*-value
Science message type^a^	1041.96	347.32	3	598	17.026	1.27e-10
Scientific language^b^	3.74	3.74	1	598	0.183	0.669
Town^c^	21.95	10.97	2	83	0.538	0.586

^a^Science message type is a factor variable with four levels corresponding to *Conventional science*, *Hero*, *Victim*, and *Victim-to-hero*.

^b^Scientific language is a factor variable with two levels corresponding to *Probability* and *Certainty* language.

^c^Town is a factor variable with three levels corresponding to *Livingston*, *Miles City*, and *Glendive*.

**Table 4:** Analysis of variance results for change in dial readings over the course of science messages for the *T*_*Net*_ and *T*_*SLR*_ statistical models.

	Sums of squares	Mean squares	Numerator degrees of freedom	Denominator degrees of freedom	*F*-value	*p*-value
***T***_***Net***_ **model**						
Segment^a^	95,674	31,891	3	2,308	82.540	< 2.2e-16
Science message type^b^	4,985	1,662	3	2,308	4.301	0.005
Scientific language^c^	87	87	1	2,308	0.226	0.634
Town^d^	507	254	2	83	0.353	0.521
Interaction between Segment and Science message type	16,458	2,351	7	2,308	6.085	4.65e-7
***T***_***SLR***_ **model**						
Segment^a^	107,525	35,842	3	2,308	83.911	< 2.2e-16
Science message type^b^	6,937	2,312	3	2,308	5.413	0.001
Scientific language^c^	375	375	1	2,308	0.879	0.349
Town^d^	477	238	2	83	0.558	0.574
Interaction between Segment and Science message type	24,412	3,487	7	2,308	8.165	7.46e-10

^a^Segment is a factor variable with four levels corresponding to *Flood definition*, *Problem framing*, *Science information*, and *Characters in action*.

^b^Science message type is a factor variable with four levels corresponding to *Conventional science*, *Victim*, *Hero*, and *Victim-to-hero*.

^c^Scientific language is a factor variable with two levels corresponding to *Probability* and *Certainty* language.

^d^Town is a factor variable with three levels corresponding to *Livingston*, *Miles City*, and *Glendive*.

### Prediction 2a

**Fig 4:**
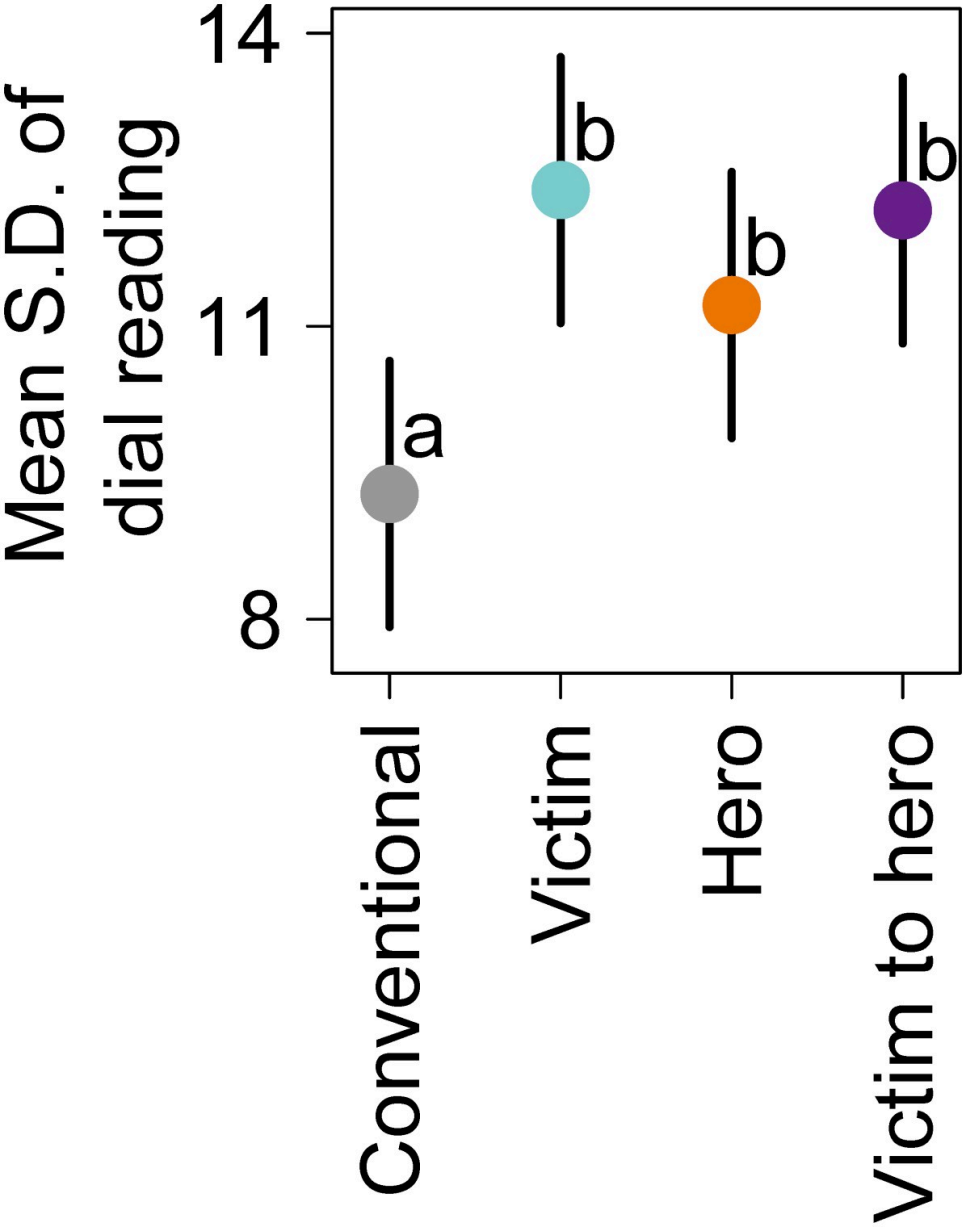
Mean standard deviation of dial readings by science message type. Point estimates and 95% confidence intervals were generated from marginal means of the linear mixed-effects models as described in Methods: Statistical analyses. Letters adjacent to point estimates denote statistically significant differences.

Prediction 2a was supported by our data. The *S*.*D*. of dial readings differed according to *Science message type* (see *Science message type* in Table 3), indicating that some *Science message types* induced more variation in affective responses than others. Importantly, the narrative science message types produced more variation in affective responses than the conventional science message types (Fig 4), supporting the hypothesis that improved risk communication via narratives may be due to a greater magnitude of affective responses compared to conventional science messages. All narrative science message types had larger *S*.*D*. in dial readings than did the conventional science message types, but no statistical differences were detected among the narrative science message types themselves **(**S1 Table. Differences in *S*.*D*. by science message type for second-by-second affective response).

### Prediction 2b

**Fig 5:**
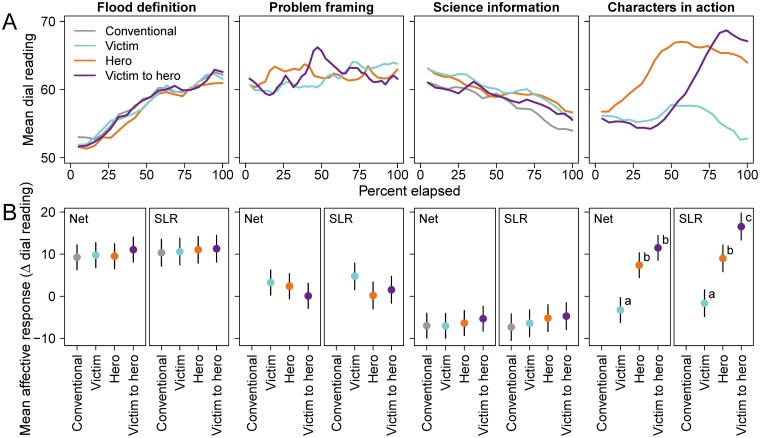
Transportation by dial readings with *T*_*Net*_ and *T*_*SLR*_. (A) Mean dial readings of participants for each science message by segment. (B) Estimates of mean change in dial readings and 95% confidence intervals by segment. Negative affective responses fall below zero and positive affective responses above zero. The calculation of transportation is indicated by Net for *T*_*Net*_ and SLR for *T*_*SLR*_ as described in Methods: Data and measures of transportation. Point estimates and 95% confidence intervals were generated from marginal means of the linear mixed-effects models as described in Methods: Statistical analyses. Letters adjacent to point estimates denote statistically significant differences.

Prediction 2b was supported by our data, but only for the *Characters in action* segment, not for the *Problem framing* segment. Overall transportation varied depending on the *Science message type*, with substantial variation among *Segments* (see *Segment*, *Science message type*, and the interaction of these two variables in Table 4). All messages start with a *Flood definition* section, which has the same text across all science messages. In Fig 5A, the mean dial readings for all *Science message types* are clustered together and show similar positive affective responses in this *Flood definition* segment. In the next segment, *Problem framing*, the affective responses vary slightly within the *Segment* by narrative character selection as the narrative characters are introduced and the problem is defined. Regardless, narrative transportation as seen by affective responses over the entire segment is fairly small for *Hero*, *Victim*, and *Victim-to-hero narratives*. When flood risk is introduced in the *Science information* segment, a general downward trend or negative affective response develops that is consistent across all the *Science message types*. However, narrative transportation for different *Characters in action* clearly reflects divergent affective responses, with the *Hero* and *Victim-to-hero narratives* inducing large positive affective responses and the *Victim narrative* inducing a small rise followed by a drop to a lower level. In other words, differences in narrative transportation among narrative *Science message types* occur mainly in the *Characters in action* segment.

Within the *Flood definition*, *Problem framing*, and *Science information* segments, the traces in dial readings depict that participants respond similarly across all *Science message types* (Fig 5A). No meaningful differences exist between the marginal means for transportation among any of the *Science message types* in these first three *Segments* (Fig 5B; S2 Table. Differences in affective response to science messages by segment). However, in the *Characters in action* segment, participants have different affective responses depending on which characters are portrayed, and these differences are statistically significant (Fig 5B; S2 Table. Differences in affective response to science messages by segment).

Within the *Characters in action* segment, narrative transportation reveals a largely negative total affective response for the *Victim narrative*, a positive response for the *Hero narrative*, and a more strongly positive response for the *Victim-to-hero narrative*. Both the *Hero narrative* and the *Victim-to-hero narrative* have significantly higher total narrative transportation in this segment than does the *Victim narrative* (Fig 5B; S2 Table. Differences in affective response to science messages by segment). The *Victim-to-hero narrative* evokes a more positive affective response than the *Hero narrative*; this difference is statistically significant in the *T*_*SLR*_ model but not the *T*_*Net*_ model (Fig 5B; S2 Table. Differences in affective response to science messages by segment).

In sum, the data support our prediction that affective responses to narrative science messages do vary depending on type of character portrayed–but only after the characters are established and the characters are acting in the drama of the story. We find that the *Hero* and *Victim-to-hero narratives* induce greater positive affective responses than does the *Victim narrative* when characters are in action, confirming that, as narrative mechanisms, characters matter in how the audience experiences the narrative.

## Threats to validity

This section discusses potential threats to the validity of the work described within this paper. Specifically, we focus on threats to construct, internal, content, and external validity [71, 72].

### Construct validity

Construct validity refers to the meaningfulness of measurements and whether both independent and dependent variables are represented correctly in the study. Although many studies use dial technology to measure affect, there are some threats to its validity as a stand-alone measure. First, we cannot know for certain the extent to which participants used the dial number readings on the Perception Analyzer^®^ to indicate affective change (up and down) versus the degree to which they used the starting point of 50 as a reference points for positive (>50) and negative (<50) affective responses. However, the dearth of scores below 50 in the aggregate is most consistent with former interpretation. Second, while there is general consensus that the structure of affect typically consists of two dimensions—valence (positive to negative) and arousal (activation) [73, 74]—dial technology measures only valence. The prompts instructing participants on dial use varied across other studies [48, 49, 65], and yet all claim to measure affective responses. In this study, our directions asked for participants to “adjust the dial upward or to the right when you like what you are hearing [and] …adjust the dial downward or to the left if you do not like what you are hearing.” To address both of these construct validity issues in the future, it may be best to use the language of valence. For example, “adjust the dial upward or to the right when you feel more positive about what you are hearing [and] …adjust the dial downward or to the left as you feel more negative about what you are hearing.” Some studies couple dial technology with physiological measures, such as heart rate and facial coding [75–77]. Although we did not use physiological measures, future studies can be designed that take these measures into account to improve construct validity.

### Internal validity

Internal validity threats refer to the possibility of having unanticipated and potentially confounding relationships between variables that may cause unreliable causes and effects. Threats can also include the omission of relevant variables. Incomplete measurement of affective responses, as discussed in the previous subsection, also constitutes a potential threat to internal validity.

### Content validity

Content validity refers to how completely the measures cover the content domain. Although we have not used physiological measures, thereby measuring arousal, we have lessened the content threats to validity by carefully measuring valence with a proven technology. Further, the topic of floods is well understood by the surveyed communities, which increases confidence in the significance of the results.

### External validity

External validity refers to the ability to generalize results beyond our case study. Although our study was conducted with three separate communities, external validity is threatened by the size of the groups participating in the research.

## Discussion

Stories are central to the human condition. Recent efforts in science communication have acknowledged the import of using story to describe scientific phenomena and knowledge [78]. Scholars in disciplines such as narratology [79], cognitive psychology [80] and hazards research [38] have identified affect as a key mechanism in narrative persuasiveness. Affect functions to transport the audience into the story. Since narratives with higher transportation are considered more effective at persuading individuals [19], risk messages that evoke more affect are likely to be more effective [20].

To link narrative transportation with character selection, our study design assumes that affective responses to narrative language serve as proxies for narrative transportation. Conditionally, our data are consistent with this assumption (particularly in the *Characters in action* segment in Fig 5). However, our data also suggest that other mechanisms may be driving affective responses to science information in the non-narrative segments of our science messages. Participants displayed some affective responses to the non-narrative language in the *Flood definition* and *Science information* segments (Fig 5). Yet, the mechanism of character selection is notably absent from these segments. Cognitive narratologists [81, 82] suggest that people may process non-narrative texts in a narrative way, perhaps through factors such as visual imagery. Other researchers suggest that confirmation/disconfirmation bias that may drive affective responses to non-narrative science statements [83–86]. Consequently, positive responses to the *Flood definition* segment and negative affective responses to the *Science message* segment could be explained by other mechanisms such as imagery or *a priori* beliefs or biases.

While our work did not test the mechanisms at work in non-narrative messages, the patterns in our data lead us to pose a new, but still untested hypothesis: confirmation/disconfirmation bias or similar mechanisms drive affective responses to non-narrative science information. This hypothesis is likely to have important implications for effective presentation of non-narrative scientific information to stakeholders. If scientific information can be presented in ways that promote positive affective responses, barriers to acceptance of new information might be lowered, improving the efficacy of risk communication.

Our study makes four additional contributions to the science of stories in risk communication. First, we show that character selection as a narrative mechanism is not a simple binary effect of presence-absence. When characters are first introduced and the problem of flooding is framed, we do not find audiences keenly engaged. However, when the drama of characters in action is presented, we see participants’ affective responses intensify in distinct directions, depending on the characters depicted. Both narratives with heroes reveal increasing positive affective responses, with a sharper and higher positive affective increase for the victim-to-hero narrative; the victim narrative dips toward negative affective responses. Thus, the drama of characters in action matters in audience experience of narrative transportation.

Second, people have larger affective responses to narrative science messages that incorporate scientific language than to conventional science messages on their own. Participants more actively turned the dial up or down during narrative science messages than during conventional science messages (Fig 4). We interpret this variation to mean they experienced more transportation when exposed to narrative science messages as compared to conventional science messages. These results advance the growing literature on narrative risk communication [47] that compares narratives and science communication by (i) embedding conventional science language in story form thereby isolating narrative effect and (ii) measuring real-time affect as the response variable.

Third, in contrast to our expectations, we find that audiences do not have larger affective responses to the language of certainty compared to the language of probability in describing flood hazards. Not only is there no difference, both presentations of flood hazard science information evoke negative affective responses across all science message types. While certainty language is posited to be more effective in communicating hazard information [52, 53], our data suggest that affective response is not the mechanism by which certainty language operates.

Fourth, this study makes important conceptual and methodological contributions. Using the Narrative Policy Framework to inform specification of narrative mechanisms is new and opens doors for further studies testing different narrative mechanisms. Additionally, we demonstrate the use of natural language processing (NLP) methods in narrative construction, which is known to engender construct validity [87]. Further, the use of dial technology to measure affective responses in real time is an innovative way to collect narrative transportation data, in contrast to measures that require summative or retrospective assessments.

Scientists want their discoveries and models to empower citizens to choose to prepare for disaster and to decrease their vulnerability to hazards. Communication strategies are key to closing the gap between scientific information about risk and peoples’ perception of and willingness to prepare for that risk. We find that characters in action matter in transporting participants, with notably positive affective responses when the target audience is depicted as the hero of his/her own story and negative affective responses with depicted as the victim. Given the potential for narrative science messages to invoke narrative transportation and improve risk communication, opportunities are ripe for future studies to assess the power of various narrative mechanisms (e.g., character selection) in science messages to shape risk perceptions and improve hazard preparedness. Results from this study suggest that a narrative-based science communication framework may serve as a *lingua franca*, or bridge language, for emergency managers and scientists to improve risk communication.

## Supporting information

S1 Protocol Interview protocol. (DOCX)

S1 Codebook Narrative Policy Framework codebook. (DOCX)

S1 Text Science messages with segments identified. (DOCX)

S1 Fig Correlation plots. (DOCX)

S1 Table Differences in *S*.*D*. by science message type for second-by-second affective response. (DOCX)

S2 Table Differences in affective response to science messages by segment. (DOCX)
