# Supplementary material for: Characters matter: How narratives shape affective responses to risk communication
Source: PLoS One. 2019 Dec 9;14(12):e0225968. doi: 10.1371/journal.pone.0225968 (PMC6901229; doi:10.1371/journal.pone.0225968)
Supplement: S1 Protocol — (DOCX) [file pone.0225968.s001.docx]

# S1 Protocol. Interview protocol

Problem Definitions & Information Sources

1. What do you value about the Yellowstone River? *[Very open ended]*
2. What do you see as problems related to the Yellowstone River, if any? *[Build on #1 response]*
3. How would you describe a flood on the Yellowstone River? What happens or what are the signs?

*[Probe for other sources of river flooding if only talk about rain and/or ice jams]*

1. What are aspects of Yellowstone River flooding that you see as problematic? *[If not answered in #2]*
2. Do you see any aspects of Yellowstone River flooding as beneficial?
3. To what extent do you think you are personally at risk from flooding on the Yellowstone River?
4. When you need information about Yellowstone River flooding, where do you go for that information?
5. What kinds of information about flooding do you pay attention to? *[If not addressed in #7]*
6. Do you wish you had other information available to you about flooding on the Yellowstone River? If so, what types or forms of information?

Flood Narratives: Heroes & Victims

1. What is your experience with river flooding events on the Yellowstone River or elsewhere? *[To the extent not covered earlier; looking for observations and adjectives]*
2. In what ways, if any, have you been harmed personally by river flooding events on the Yellowstone River or elsewhere? *[Victim (i.e., someone harmed)]*
3. In what ways, if any, has your community been harmed by river flooding events on the Yellowstone River or elsewhere? *[Victim]*
4. Who, if anybody, helped you personally to prepare for or recover from flooding events? How did they help? *[Probe for both before and after as necessary; heroes & decisions]*
5. Who, if anybody, helped your community to prepare for or recover from flooding events? How did they help? *[Probe for both before and after as necessary; heroes & decisions]*
