# Supplementary material for: Characters matter: How narratives shape affective responses to risk communication
Source: PLoS One. 2019 Dec 9;14(12):e0225968. doi: 10.1371/journal.pone.0225968 (PMC6901229; doi:10.1371/journal.pone.0225968)
Supplement: S1 Codebook — (DOCX) [file pone.0225968.s002.docx]

# S1 Codebook. Narrative Policy Framework codebook

1. Characters can be a human or non-human entity.
   1. Hero is a parent node: an entity who is cast to solve the problem
   2. Victim is a parent node: an entity who fears harm or suffers from the problem
   3. Villain is a parent node: an entity who causes the problem in the first place

Children nodes for the above parent nodes will be specific names (often *in vivo*), e.g., FEMA or the Yellowstone River.

1. Preparation Decisions are what people identify as actionable items.
   1. Actual decisions is the parent node: identifiable action items
   2. Could-do decisions is the parent node: identifiable action items

Children nodes for the above parent nodes will be individual/family decisions and community/neighbor decisions.
