# Supplementary material for: Characters matter: How narratives shape affective responses to risk communication
Source: PLoS One. 2019 Dec 9;14(12):e0225968. doi: 10.1371/journal.pone.0225968 (PMC6901229; doi:10.1371/journal.pone.0225968)
Supplement: S1 Text — (DOCX) [file pone.0225968.s003.docx]

# S1 Text. Science messages with segments identified

Conventional science message with probability language

*[Flood definition]* Flooding occurs when water flows over land that is usually dry. Flooding can result from rain, snowmelt, and high flows in waterways. Flooding can also result from waterways being blocked by debris or ice and from infrastructure failures.

*[Scientific information: Probability language]* A flood large enough to qualify as a 100-year flood has a 1% chance of happening in any single year. Yet the chance of flooding also adds up over time. For example, a 100-year flood has a 26-percent chance of occurring in any 30-year period.

Hero narrative science message with probability language

*[Flood definition]* Flooding occurs when water flows over land that is usually dry. Flooding can result from rain, snowmelt, and high flows in waterways. Flooding can also result from waterways being blocked by debris or ice and from infrastructure failures.

*[Hero problem framing]* In Montana, good neighbors like you help when Yellowstone floodwaters rise. While many feel protected by the local dike, people also understand and respect the power of this river. Engineers provide the benefits of technical assistance to reduce flood risks. Yet, in the face of a large flood event, even the best-engineered solutions may not work. Under extreme flood conditions, the river could over-top your levee or bank.

*[Scientific information: Probability language]* A flood large enough to qualify as a 100-year flood has a 1% chance of happening in any single year. Yet the chance of flooding also adds up over time. For example, a 100-year flood has a 26-percent chance of occurring in any 30-year period.

*[Hero character in action]* Working together with your local emergency responders, you can think about and begin to implement individual and community strategies before a disaster occurs. By trying to protect from damages of extreme flooding, you will have really helped in a big way.

Victim narrative science message with probability language

*[Flood definition]* Flooding occurs when water flows over land that is usually dry. Flooding can result from rain, snowmelt, and high flows in waterways. Flooding can also result from waterways being blocked by debris or ice and from infrastructure failures.

*[Victim problem framing]* Many homeowners and businesses along the Yellowstone River are concerned with having to pay a lot of money for insurance based on the latest federal flood map. While most respect the power of this river, your house or your friend’s house and the economy of local business could be vulnerable. Even with engineered solutions to reduce flood worries, problems can happen quickly. Under extreme flood conditions, the river could over-top, leaving properties damaged and companies hit with expensive repair costs.

*[Scientific information: Probability language]* A flood large enough to qualify as a 100-year flood has a 1% chance of happening in any single year. Yet the chance of flooding also adds up over time. For example, a 100-year flood has a 26-percent chance of occurring in any 30-year period.

*[Victim character in action]* You, your friends, and your neighbor could be harmed or wiped out by high post-flood premiums and loss of valuable assets such as cattle and houses. Without preparation, your town could be lost as it faces difficult and sad times.

Victim-to-hero narrative science message with probability language

*[Flood definition]* Flooding occurs when water flows over land that is usually dry. Flooding can result from rain, snowmelt, and high flows in waterways. Flooding can also result from waterways being blocked by debris or ice and from infrastructure failures.

*[Victim-to-hero problem framing]* Many homeowners and businesses along the Yellowstone River are concerned with having to pay a lot of money for insurance based on the latest federal flood map. Yet, in Montana, good neighbors like you help when Yellowstone floodwaters rise. While many feel protected by the local dike, your house or your friend’s house and the economy of local business could be vulnerable, as even the best-engineered solutions may not work. Under extreme flood conditions, the river could over-top, leaving properties damaged and companies hit with expensive repair costs.

*[Scientific information: Probability language]* A flood large enough to qualify as a 100-year flood has a 1% chance of happening in any single year. Yet the chance of flooding also adds up over time. For example, a 100-year flood has a 26-percent chance of occurring in any 30-year period.

*[Victim-to-hero character in action]* Without preparation, your town could be lost, as it faces difficult and sad times. Working together with your local emergency responders, you can think about and begin to implement individual and community strategies before a disaster occurs.

Conventional science message with certainty language

*[Flood definition]* Flooding occurs when water flows over land that is usually dry. Flooding can result from rain, snowmelt, and high flows in waterways. Flooding can also result from waterways being blocked by debris or ice and from infrastructure failures.

*[Scientific information: Certainty language]* The potential for flooding along waterways is greater than commonly understood. Towns are often situated on sand and gravel that was deposited along waterways during past large floods. Similar floods will occur in the future, flooding these towns.

Hero narrative science message with certainty language

*[Flood definition]* Flooding occurs when water flows over land that is usually dry. Flooding can result from rain, snowmelt, and high flows in waterways. Flooding can also result from waterways being blocked by debris or ice and from infrastructure failures.

*[Hero problem framing]* In Montana, good neighbors like you help when Yellowstone floodwaters rise. While many feel protected by the local dike, people also understand and respect the power of this river. Engineers provide the benefits of technical assistance to reduce flood risks. Yet, in the face of a large flood event, even the best-engineered solutions may not work. Under extreme flood conditions, the river could over-top your levee or bank.

*[Scientific information: Certainty language]* The potential for flooding along waterways is greater than commonly understood. Towns are often situated on sand and gravel that was deposited along waterways during past large floods. Similar floods will occur in the future, flooding these towns.

*[Hero character in action]* Working together with your local emergency responders, you can think about and begin to implement individual and community strategies before a disaster occurs. By trying to protect from damages of extreme flooding you will have really helped in a big way.

Victim narrative science message with certainty language

*[Flood definition]* Flooding occurs when water flows over land that is usually dry. Flooding can result from rain, snowmelt, and high flows in waterways. Flooding can also result from waterways being blocked by debris or ice and from infrastructure failures.

*[Victim problem framing]* Many homeowners and businesses along the Yellowstone River are concerned with having to pay a lot of money for insurance based on the latest federal flood map. While most respect the power of this river, your house or your friend’s house and the economy of local business could be vulnerable. Even with solidly engineered solutions to reduce flood worries, problems can happen quickly. Under extreme flood conditions, the river could over-top, leaving properties damaged and companies hit with expensive repair costs.

*[Scientific information: Certainty language]* The potential for flooding along waterways is greater than commonly understood. Towns are often situated on sand and gravel that was deposited along waterways during past large floods. Similar floods will occur in the future, flooding these towns.

*[Victim character in action]* You, your friends, and your neighbor could be harmed or wiped out by high post-flood premiums and loss of valuable assets such as cattle and houses. Without preparation, your town could be lost as it faces difficult and sad times.

Victim-to-hero narrative science message with certainty language

*[Flood definition]* Flooding occurs when water flows over land that is usually dry. Flooding can result from rain, snowmelt, and high flows in waterways. Flooding can also result from waterways being blocked by debris or ice and from infrastructure failures.

*[Victim-to-hero problem framing]* Many homeowners and businesses along the Yellowstone River are concerned with having to pay a lot of money for insurance based on the latest federal flood map. Yet, in Montana, good neighbors like you help when Yellowstone floodwaters rise. While many feel protected by the local dike, your house or your friend’s house and the economy of local business could be vulnerable, as even the best-engineered solutions may not work. Under extreme flood conditions, the river could over-top, leaving properties damaged and companies hit with expensive repair costs.

*[Scientific information: Certainty language]* The potential for flooding along waterways is greater than commonly understood. Towns are often situated on sand and gravel that was deposited along waterways during past large floods. Similar floods will occur in the future, flooding these towns.

*[Victim-to-hero character in action]* Without preparation, your town could be lost as it faces difficult and sad times. Working together with your local emergency responders, you can think about and begin to implement individual and community strategies before a disaster occurs.
