# Supplementary material for: Characters matter: How narratives shape affective responses to risk communication
Source: PLoS One. 2019 Dec 9;14(12):e0225968. doi: 10.1371/journal.pone.0225968 (PMC6901229; doi:10.1371/journal.pone.0225968)
Supplement: S1 Fig — (DOCX) [file pone.0225968.s004.docx]

# S1 Fig. Correlation plots

#
