# Supplementary material for: Characters matter: How narratives shape affective responses to risk communication
Source: PLoS One. 2019 Dec 9;14(12):e0225968. doi: 10.1371/journal.pone.0225968 (PMC6901229; doi:10.1371/journal.pone.0225968)
Supplement: S1 Table — (DOCX) [file pone.0225968.s005.docx]

# S1 Table. Differences in *S.D.* by Science Message Type for Second-by-Second Affective Response

# Differences in Standard Deviation by Science Message Type for Second-by-Second Affective Response

|  | **Difference estimate**^a,b^ | **t-ratio** | ***p*-value** |
| --- | --- | --- | --- |
| Hero – conventional | 1.935 | 3.973 | 0.001 |
| Victim – conventional | 3.112 | 6.389 | < 0.001 |
| Victim-to-hero – conventional | 2.905 | 5.964 | < 0.001 |
| Victim – hero | 1.177 | 2.416 | 0.075 |
| Victim-to-hero – hero | 0.970 | 1.991 | 0.193 |
| Victim – victim-to-hero | 0.207 | 0.425 | 0.974 |

^a^Estimates are differences in standard deviation based on the type of message. For example, the first row compares the hero narrative against the conventional science message. Difference estimates are averaged across probability/certainty language and across towns.

^b^For each pairwise comparison: standard error = 0.487; degrees of freedom = 598
