# Supplementary material for: Characters matter: How narratives shape affective responses to risk communication
Source: PLoS One. 2019 Dec 9;14(12):e0225968. doi: 10.1371/journal.pone.0225968 (PMC6901229; doi:10.1371/journal.pone.0225968)
Supplement: S2 Table — (DOCX) [file pone.0225968.s006.docx]

# S2 Table. Differences in affective response to science messages by segment

| **Differences in Affective Response to Science Messages by Segment** | | | | | | |
| --- | --- | --- | --- | --- | --- | --- |
|  |  | **DV:** T_Net_^a^ | |  | **DV:** T_SLR_^b^ | |
|  | **Difference estimate** ^c,d^ | ***t*-ratio** | ***p*-value** | **Difference estimate** ^c,e^ | ***t*-ratio** | ***p*-value** |
| **Segment 1:**  **Flood definition** |  |  |  |  |  |  |
| Hero – conventional | 0.279 | 0.132 | 0.999 | 0.708 | 0.317 | 0.989 |
| Hero – victim | -0.233 | -0.110 | 1.000 | 0.431 | 0.193 | 0.997 |
| Hero – victim-to-hero | -1.547 | -0.730 | 0.885 | -0.253 | -0.114 | 1.000 |
| Conventional – victim | -0.512 | -0.241 | 0.995 | -0.277 | -0.124 | 0.999 |
| Conventional – victim-to-hero | -1.826 | -0.861 | 0.825 | -0.961 | -0.431 | 0.973 |
| Victim – victim-to-hero | -1.314 | -0.620 | 0.926 | -0.684 | -0.307 | 0.990 |
| **Segment 2:**  **Problem definition** |  |  |  |  |  |  |
| Hero – victim | -0.861 | -0.406 | 0.977 | -4.527 | -2.031 | 0.177 |
| Hero – victim-to-hero | 2.297 | 1.083 | 0.700 | -1.352 | -0.607 | 0.930 |
| Victim – victim-to-hero | 3.157 | 1.489 | 0.444 | 3.175 | 1.425 | 0.484 |
| **Segment 3:**  **Science information** |  |  |  |  |  |  |
| Hero – conventional | 0.611 | 0.288 | 0.992 | 2.178 | 0.978 | 0.762 |
| Hero – victim | 0.669 | 0.315 | 0.989 | 1.238 | 0.555 | 0.945 |
| Hero – victim-to-hero | -1.041 | -0.491 | 0.961 | -0.457 | -0.205 | 0.997 |
| Conventional – victim | 0.058 | 0.027 | 1.000 | -0.941 | -0.422 | 0.975 |
| Conventional – victim-to-hero | -1.652 | -0.779 | 0.864 | -2.635 | -1.182 | 0.638 |
| Victim – victim-to-hero | -1.709 | -0.806 | 0.851 | -1.694 | -0.760 | 0.872 |
| **Segment 4:**  **Characters in action** |  |  |  |  |  |  |
| Hero – victim | 10.616 | 5.009 | 0.001 | 10.622 | 4.766 | < 0.0001 |
| Hero – victim-to-hero | -4.111 | -1.939 | 0.212 | -7.546 | -3.386 | 0.004 |
| Victim – victim-to-hero | -14.727 | -6.948 | 0.001 | -18.168 | -8.152 | < 0.0001 |

^a^T_Net_ is the net difference between the affective response score at the end of a segment for an individual and the affective response score at the start of that same segment.

^b^T_SLR_ is the mathematical product of slope and message duration estimated from fitting a simple linear regression for each segment at the individual level.

^c^A negative estimate indicates that the latter message produced a more positive change in response. Results are averaged across probability and certainty language. Results are also averaged across respondent towns. The standard error is 2.12 for all estimates for the first dependent variable and is 2.23 for the second dependent variable. The Tukey method for comparing a family of four estimates was used in adjusting the *p*-values.

^d^For each pairwise comparison for T_Net_: standard error = 2.12 and degrees of freedom = 2308

^e^For each pairwise comparison for T_SLR_: standard error = 2.23 and degrees of freedom = 2308
